# Dr. Edwin T. M. Hurlbut

**Published:** 1915-06

**Authors:** 


**Dr. Edwin T. M. Hurlbut,** Buffalo 1867, died at the Veterans' Home in Napa Co., Calif., April 12, aged 87. He served in the 24th N. Y. Artillery.

